# Role of Illumination and Light Colour Temperature in the Preference Behaviour of Weaned Piglets

**DOI:** 10.3390/ani15213116

**Published:** 2025-10-27

**Authors:** Sven Götz, Klaus Reiter, Monika Wensch-Dorendorf, Eberhard von Borell, Camille M. C. Raoult

**Affiliations:** 1 Institute of Agricultural and Nutritional Sciences, Department of Animal Husbandry and Ecology, Martin-Luther-University Halle-Wittenberg, Theodor-Lieser-Str. 11, 06120 Halle (Saale), Germany; eberhard.vonborell@landw.uni-halle.de; 2 Bavarian State Office for Agriculture, Institute for Agricultural Engineering and Animal Husbandry, Prof.-Dürrwaechter-Platz 5, 85586 Poing-Grub, Germany; klaus.reiter@lfl.bayern.de; 3 Institute of Agricultural and Nutritional Sciences, Department of Biometrics and Agricultural Informatics, Martin-Luther-University Halle-Wittenberg, Karl-Freiherr-von-Fritsch-Str. 4, 06120 Halle (Saale), Germany; monika.dorendorf@landw.uni-halle.de; 4 Department of Agroecology and Environment, ISARA, 23 Rue Jean Baldassini, 69364 Lyon Cedex 07, France

**Keywords:** illuminance, colour temperature, weaned piglets, preference behaviour, light-emitting diode (LED), pig housing

## Abstract

**Simple Summary:**

Light plays an important role in the behaviour, health and productivity of livestock. While the effects of day length on pigs—particularly in the context of reproduction—have been studied, little is known about how light intensity and colour temperature influence their behaviour. In this study, the impact of different brightness levels (80 lux and ~0 lux) and two-colour temperatures (bluish light at 6500 Kelvin and reddish light at 3000 Kelvin) was examined. The piglets were free to move between the different lighting conditions and were continuously observed by cameras, with their behaviour analysed afterwards. Initially, piglets in the first batch preferred darkened areas, whereas, in batch two, they stayed in the illuminated pens, especially when the colour temperature was 3000 Kelvin. However, this changed as the animals grew older, with the piglets’ preference shifting towards the darker areas until, by the end of the study, no clear preference could be detected. While the darkened areas remained largely clean, the area with the 6500 Kelvin colour temperature was more frequently soiled with faeces. These findings suggest that pigs respond to different lighting conditions and may benefit from lighting concepts aligned with their natural behaviour. This knowledge can help improve both animal welfare and the design of pig housing in swine production.

**Abstract:**

This study investigated the preference behaviour of 24 four-week-old weaned piglets under different lighting conditions (0 lux with 0 Kelvin vs. 80 lux with 3000 Kelvin vs. 6500 Kelvin). Two trials with 12 piglets each were conducted over five weeks in a room with four interconnected pens, allowing free movement between the pens. Pens A and B were nearly dark (~0 lux), while pen C (80 lux, 3000 Kelvin) and pen D (80 lux, 6500 Kelvin) were illuminated. On three days in weeks 1, 3 and 5, behaviour (lying, eating and activity) was recorded using video observations and a 5 min time sampling method. Cleanliness was also monitored daily. In the first week, piglets in the first batch preferred the darkened pens, whereas piglets in the second batch preferred illuminated pens, especially when the colour temperature was 3000 Kelvin. By the third week, piglets in the second batch now preferred darker areas. In the fifth week, the piglets spent more time in the dark in the mornings and evenings but showed no preference for colour temperature. The darkened pens remained mostly clean, whereas pen D, which had a light colour temperature of 6500 Kelvin, was the most soiled. The results show that piglet behaviour changes with age and the time of day, suggesting that lighting concepts can be adapted to improve both animal welfare and pen hygiene.

## 1. Introduction

Both direct and indirect effects of light have been recorded in different animal species. In pigs, light affects weight development, feed intake, sexual behaviour, reproduction and general behaviour [1,2,3,4,5,6,7,8,9]. Alongside these positive effects, light can also have negative effects on the health and behaviour of pigs. Excessive exposure to light, flickering light sources or permanent lighting can lead to increased aggression, weight loss and eye damage in pigs [10,11,12]. These harmful effects have been demonstrated in humans and in other animal species, including mice and chickens [13,14,15,16]. Scaillierez et al. found no negative effect of light intensity on the health of piglets at different light levels (45 lux (lx) low intensity, 198 lx medium intensity and 968 lx high intensity) [17]. Despite the different effects of light on pigs, legal regulations remain rare and often only regulate the minimum illuminance (also referred to as light intensity) and duration of light phases. The recommendations for light in pig husbandry range from 20 lx to 100 lx or higher [18,19,20,21]. However, there is no information about the colour temperature or the flicker-free nature of the light sources used, as is regulated in the German animal welfare regulations for poultry, for example [20].

Preference tests, in which growing pigs were offered a choice between different illuminance levels, have given contradictory results. Taylor et al. [21] observed that growing piglets showed a preference for resting in dark compartments (<1 lx) and a tendency to defaecate in brighter compartments (400 lx). Based on their results, and due to pigs’ natural exploratory behaviour, Taylor et al. assumed that pigs prefer a range of lighting conditions rather than a fixed light environment [7,21]. Indeed, the intensity of natural daylight varies greatly over the seasons and within a day, from several thousand lux for direct sunlight to 1000–2000 lx (and between 5000 and 6500 Kelvin (K)) for typical midday daylight, to 100–400 lx at sunrise, to less than 5 lx under thick storm clouds and to less than 1 lx by moonlight [22]. Götz et al. [3] showed that four-week-old piglets had an initial preference for brightly lit pens (600 lx), changing their preference to darkened pens (close to 0 lx) within three weeks. Later, in the fifth week of the experiment, no preference could be determined. Similar behaviours were observed in a study by Tanida et al. [22], where one-week-old piglets avoided darkness (5 lx) and tended to move to the brightly lit parts (2100 lx) of the test area. In contrast, Christison et al. [23] found no general preference in piglets for lit over unlit resting areas. It is assumed that the different ages (1 week to 6 weeks) of the animals tested in the trials, as well as the lighting conditions the sows were accustomed to in the farrowing pen, influenced the results [23,24].

It is known that pigs perceive their environment primarily through touch and smell, with vision playing a subordinate role. Compared to humans, the eyesight of pigs is considered very limited, and the area of sharp vision is restricted to a small field of view [25]. Even though pigs, unlike humans, have dichromatic vision only, they can distinguish between different colours [4,7,9,26,27,28]. Studies on colour preference by Klocek et al. [29] showed that piglets (aged between 6 and 8 weeks) visited blue feeding troughs more frequently and for longer periods than yellow comparative models. This is attributed to the better colour perception of the troughs by the pigs. Deligeorgis et al. [9] found in their studies on the colour preference of newborn piglets (at the time of the study, the piglets were approximately 48 h old) that the piglets consumed more water from red and blue drinkers than from green ones. A gender-specific difference in colour preference was also demonstrated; males preferred red, while females preferred blue.

Götz et al. [2] showed that growing piglets have a preference for a colour temperature of 3000 K (reddish light) compared to 6500 K (bluish light) when exposed to 80 lx during the day and 0.3 lx at night. This preference was most evident in piglets’ resting behaviour, as they spent most of their time lying down below the 3000 K light.

In preference tests, this preference for “warmer” reddish light (2500 K or 4000 K at 91 lx) was also observed in mice [30]. In the study conducted by Kapogiannatou et al. [31], it was demonstrated that mice exhibited reduced stress levels at a colour temperature of 2500 K and an illumination intensity of 60 lx in comparison to the levels observed at a colour temperature of 4000 K and an illumination intensity of 60 lx. However, that study found no significant impact on locomotor activity.

In a study by Paggi et al. [32], piglets (aged 3–5 days) did not show any preference for reddish lighting. The animals preferred the green-lit areas and avoided red and blue lighting. However, the colours were always tested against a neutral white light and not against each other.

The present study should be regarded as a pilot study to investigate piglets’ preference between two illuminance levels (i.e., close to 0 lx or 80 lx) combined with three different colour temperatures for the light-emitting diode (LED) lit pens (i.e., 3000 K or 6500 K at 80 lx or 0 K at ~0 lx). As the behaviour of piglets was found to differ according to either the illuminance or the colour temperature independently, in the current study, the piglets’ choices when exposed to both these stimulants simultaneously were evaluated. It was hypothesised that piglet preferences observed in previous studies—namely, an initial strong preference for the 3000 K, 80 lx illuminated pen and, with increasing age, for the darkened compartments, particularly when lying down—would also be evident in the present study [2,3].

If this is indeed the case and the piglets distribute their activities in different areas of the pen according to the lighting conditions, recommendations could be made regarding the light to provide pigs with clearly differentiated functional areas depending on their age and behavioural needs. Thus, piglets’ eating, lying and activity preference behaviours were evaluated by recording the percentage of piglets under each lighting condition in each pen (A = 0 K and ~0 lx, B = 0 K and ~0 lx, C = 3000 K and 80 lx, D = 6500 K and 80 lx) using a 5 min time sampling method. In addition, the degree of soiling in each pen was recorded to assess the pigs’ lighting preference when defaecating.

## 2. Materials and Methods

### 2.1. Animals

A total of 24 four-week-old weaned piglets (12 females and 12 castrated males of the Large-White × Landrace × Piétrain cross breed) were housed in two successive batches in the preference test room of the Martin-Luther-University of Halle-Wittenberg (Halle, Germany) for five weeks each. The piglets were bought directly after weaning from the WM Agrar GmbH & Co. KG (conventional pig producer; Wallhausen, Germany), where they had only experienced 80 lx light illuminance in pens with windows (natural light; no more information about the lighting was available).

The first contact between the piglets took place during transport, an event that was unavoidable due to the supplier’s management. Therefore, the influence of this social component on subsequent behaviour cannot be taken into consideration.

On the day the first batch of 12 piglets arrived, which was also the first day of the trial, the piglets were weighed individually, clearly marked and distributed evenly by sex when being placed in the four pen compartments. The piglets weighed on average 8.44 ± 1.1 kg (females 8.27 ± 1.0 kg; castrated males 8.61 ± 1.0 kg). Data collection started when the last animal had been placed in the experimental pen, followed by the opening of all the passageways between the pen compartments. This allowed the piglets to move freely between the four pens from the start of the experiment and thus to choose between the different lighting situations. Animals were not acclimatised to the testing environment beforehand.

Throughout the experiment, the piglets had ad libitum access to pellet food (Mini Start, Denkapig in the early days and FA I later, Agravis, Querfurt, Germany) from automatic feeders and water from nipple drinkers (a water bowl for the first few days until it was ensured that all animals were using the nipple drinkers). Each pen was equipped with one feeder and one drinker. The routine work in the test room, such as animal inspection, refilling the feeders and cleaning of the pens, took place daily between 08:00 and 10:00. To avoid effects of habituation and behavioural changes (e.g., avoidance of compartments), the order of cleaning was changed. No data were collected during routine work, as the animals seemed to be disturbed by the work. The routine work also included remarking of the pigs for identification purposes.

### 2.2. Ethical Statement

The experiment was notified to the Veterinary Affairs of the Administration Office of Saxony-Anhalt, Germany, but no additional permission was required with regard to the Animal Welfare Act (§7 para. 2 TierSchG), as the university had general authorisation from the German Veterinary Office (No. TS 17/2018) to house pigs for experimental purposes in the preference test room of the Martin-Luther-University of Halle-Wittenberg as long as no measures causing pain, suffering or injury to these animals were performed.

### 2.3. Experimental Design

The preference test room used for this experiment was located in the Institute of Agricultural and Nutritional Sciences of the Martin-Luther-University of Halle-Wittenberg. The test room setup consisted of four identically furnished pens in one compartment connected by 47 cm × 35 cm large passageways. This setup allowed the piglets to move freely between the different pens throughout the entire experiment to choose their preferred lighting situation. The passageways were obscured by three hanging opaque black polyvinyl chloride (PVC) strips (55 cm high × 20 cm wide × 0.5 cm thick each; Thermonet Trade, Mannheim, Germany).

**Figure 1:**
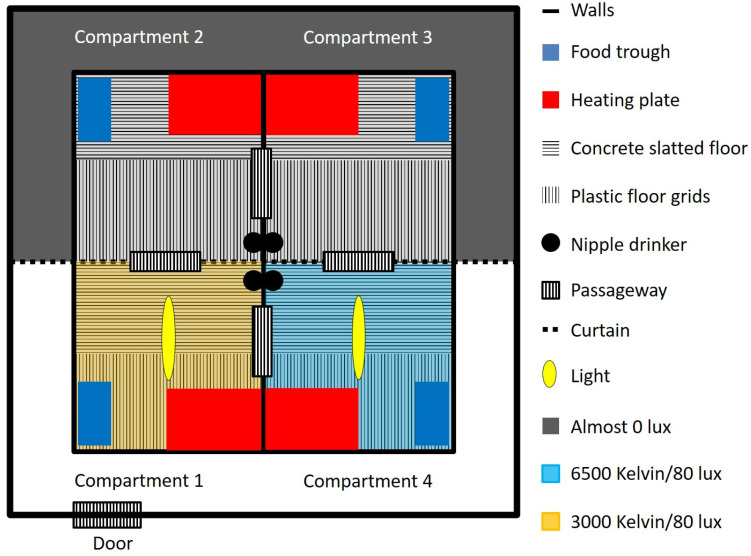
Design of the preference test room and the four lighting situations in each pen of the first batch. (Pen A,B) = ~0 lux, 0 Kelvin; (Pen C) = 80 lux, 3000 Kelvin; (Pen D) = 80 lux, 6500 Kelvin.

The lighting situations in the compartments were given over the entire test duration (24 h/7 days/5 weeks). Two pens were permanently close to 0 lx and 0 K (pen A and pen B), one pen was permanently illuminated with 80 lx and a colour temperature of 3000 K (pen C), and the last pen was permanently illuminated with 80 lx and 6500 K (pen D; Figure 1).

The passageways between the pens were open from the start of the experiment, allowing the piglets to choose their preferred combination of illuminance and colour temperature from the outset. To ensure the different lighting conditions within each pen, a wooden scaffold was installed in the middle of the room and covered with black, opaque fabric (Sunblock blackout fabric; Tuchler; Vienna, Austria) to prevent light entering from the illuminated pens (C, D).

Each pen had a total area of 4.17 m^2^ (i.e., 1.67 m × 2.49 m = 4.17 m^2^/12 piglets = 0.39 m^2^/piglet in each pen and 1.39 m^2^/piglet in the whole compartment) and consisted of a concrete slatted floor covering half of the surface, an embedded heating plate (40 cm × 60 cm, MIK International THERMO E, MIK International, Ransbach-Baumbach, Germany; red rectangle in Figure 1) and a food trough (28 cm × 55 cm × 65 cm OK Plast, Horsens, Denmark; blue rectangle in Figure 1) fixed to the wall, and, on the remaining half, plastic floor grids (MIK International Rubin, MIK International, Ransbach-Baumbach, Germany) and a nipple drinker were installed (the flow rate was adjusted to the growth of the piglets) (Figure 1).

The housing conditions complied with the minimum space requirements specified in the German Animal Welfare Regulations for farm animals (i.e., 0.15 m^2^/piglet up to 10 kg live weight, 0.20 m^2^/piglet up to 20 kg, and 0.35 m^2^/piglet up to 30 kg) as well as with those of the EU Directive on the protection of animals used for scientific purposes (i.e., 0.25 m^2^/piglet up to 10 kg live weight, 0.35 m^2^/piglet up to 20 kg, and 0.50 m^2^/piglet up to 30 kg), taking into account the total space available in the pen.

As enrichment materials, a sisal rope (attached to the framework) and a chain with a piece of softwood (in the middle of the pen) were available in each pen. For the first batch, pen C was permanently illuminated (24 h) with 80 lx and a colour temperature of 3000 K, while pen D was permanently illuminated with 80 lx and 6500 K. The two opposite pens (A and B) remained dark, close to 0 lx. To minimise the possible effect of the position of the pen on the behaviour of the pigs, the lighting conditions in the test room were reversed for the second batch so the darkened pen compartments were near the entrance of the test room (Figure 1). For reasons of clarity and comprehensibility, the labelling of the pens (A-D) was maintained throughout the experiment, even though the lighting scheme in the pens was changed.

Before the test started, the installation height of the LED lighting for each pen was determined using the DIALux evo 9.1 software (DIAL GmbH, Luedenscheid, Germany). For this purpose, the software created a digital model of the test room and experimental setup and then, when given the target value of 80 lux, the program calculated the required specifications for the LED lighting and the optimum installation height. A Schuch 161-161 LED lighting (Adolf Schuch GmbH, Worms, Germany) was used, mounted centrally 1.35 m above the pen floor on a wooden beam.

Illuminance in each pen was checked using a luxmeter (Mavolux 5032B Luxmeter, Gossen Foto-und Lichtmesstechnik GmbH, Nuremberg, Germany). For this purpose, a measurement grid was laid out in each pen (20 cm × 20 cm), and the illuminance of the LED at each measurement point was determined at a height of 25 cm (the approximate height of the pigs’ eyes). Based on these measurement results, the installation height of the LED was optimised.

An average uniformity of the illuminance of g1 = 0.92 (i.e., the value of the ratio between the minimum to average measured illuminance of the measurement grid) was achieved for the illuminated pens. The uniformity achieved was higher than the minimum value of g1 = 0.6 required by the German and European standard DIN EN 12464-1 (i.e., requirements for the lighting of indoor workplaces, taking into account the visual performance of persons with normal or normally corrected vision and visual comfort [33]) and used here as a guideline.

For the darkened compartments, an average uniformity of the illuminance of g1 = 0.42 was achieved. However, the uniformity was considered sufficient, as it only affected the front part of the pens (the line of the measurement grid nearest the PVC strips). The black PVC strips reduced light penetration into the darkened compartments but could not completely prevent it, especially when a pig crossed the strips or touched them. As a result, the measurement points in areas near the passageway had slightly higher illuminance values (0.05 lx, which corresponds approximately to a starry night without moonlight) than the measurement points at the back of the pens, which were close to 0 lux. This caused less consistencyin the measurements in the darkened pens.

### 2.4. Measurements

To investigate the influence of the lighting conditions on the preference behaviour of weaned piglets, four video cameras were installed above each pen (Monacor HDCAM 630, Monacor International GmbH & Co. KG, Bremen, Germany; a 2-megapixel HD-SDI colour camera with day/night function and 2.8–12 mm varifocal lens). This equipment allowed a full view of the pens to observe the piglets at any location in the test room. The cameras continuously recorded the piglets’ location and behaviour. Video recordings were stored on a digital recorder (EPHD 08 Everfocus Electronics Corp., New Taipei, Taiwan) and transferred to digital storage media for later analysis. Behavioural observations were paused between 08:00 and 10:00 during routine stable work.

Video recordings of three days (Tuesday, Saturday and Sunday) during the experiment’s first, third and fifth week were analysed using a 5 min time sampling method. For this purpose, the video footage of the recorded 24 h periods were stopped every 5 min using the video player EFPlayer HD (version 1.0.7.9; Everfocus Electronics Corp., New Taipei, Taiwan) and each behaviour and the position of the animals were recorded according to the ethogram described below.

The ethogram distinguished between “lying”, “feeding”, “drinking,” “moving” and “active” animals. A pig was considered “lying” if it was either in a sternal or lateral position. No additional distinction was made concerning whether an animal was asleep or lying down. The animals were counted as “eating” if their heads were visible up to their ears above the feeder or if they were chewing. If an animal had its head above the trough, it was counted as “feeding” even without visible chewing movements since the camera’s angle did not allow further distinction. “Drinking” was recorded if a pig had its snout on the nipple drinker at the time of analysis. However, there was no distinction as to whether the animal was at the nipple drinker to drink water or for other reasons such as exploratory or play behaviour. There was also no recording of the quantity of water absorbed.

Pigs were classified as “moving” if they were in directed motion at the moment of observation. The behaviour “active” contained all actions that did not fall into the other behavioural categories (e.g., interactions with other pigs or with the pen). Due to the limited occurrences, the observed “drinking” and “moving” behaviours were later combined with the “active” behaviour for the statistical analyses.

These behaviours, along with the location (3000 K (C)/6500 K (D)/~0 lx (A)/~0 lx (B)), were noted in an Excel spreadsheet according to a binary system (i.e., 1 = performing/present, 0 = not performing/absent). One animal could only perform one action per observation period. This action could also only be performed under one lighting condition. This methodology was chosen because it was used and proven to be effective in previous studies, thus allowing comparability with previous results in this testing setup [2,3].

To determine the preferred lighting condition for eliminating faeces and urine, the cleanliness of the pens was evaluated. A daily assessment was made based on the methodology of the Bavarian State Institute for Agriculture for measuring the cleanliness of partially slatted floor pens of fattening pigs [34]. Due to the design of the test room, however, only the solid excrements of the piglets could be evaluated, as any liquid components were immediately discharged through the slatted floor and into the drain.

The cleanliness of the pens was scored from 0 to 4 as follows: 0 = not soiled, i.e., a clean pen with no faecal contamination; 1 = very slightly soiled, i.e., visible individual piles of faeces; 2 = slightly soiled, i.e., slightly visible soiling with faeces covering less than 25% of the floor; 3 = moderately soiled, i.e., medium visible soiling with faeces covering more than 25% and up to 50% of the floor; and 4 = heavily soiled, i.e., high degree of soiling with faeces covering more than 50% of the floor.

**Figure 2:**
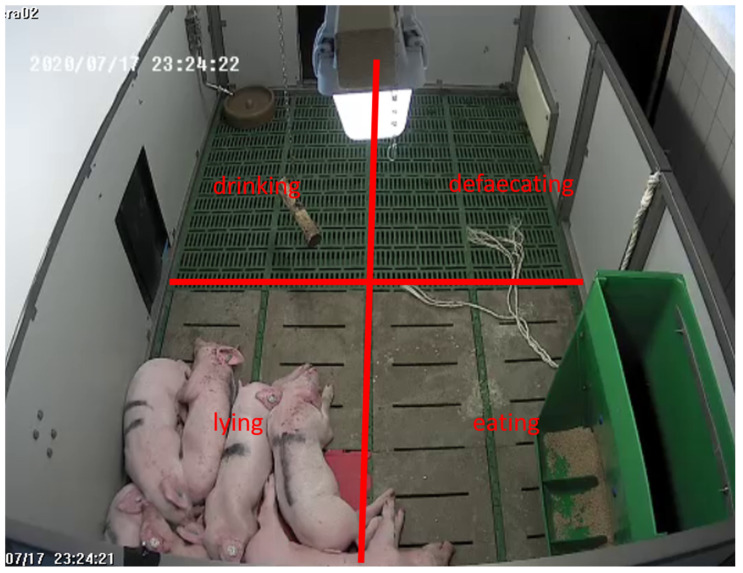
Virtual division (red lines) into the four functional areas, defaecating, drinking, eating and lying, as illustrated for one pen.

For visual assessment, each pen was divided into four equal squares (Figure 2). The classification was made according to the function of the area: the so-called “defaecation area” with the highest proportion of slatted floor, “drinking area” around the nipple drinker, “feeding area” at the trough and the “lying area” around the heating plate.

### 2.5. Statistical Analyses

Data were prepared and checked for unplausible or missing data using Microsoft Excel (Microsoft Corporation, Redmont, WA, USA). Subsequent statistical analyses were performed using Statistical Analysis System 9.4 (SAS Institute Inc., Cary, NC, USA). All behavioural observations of one day were summed up into two-hour segments to facilitate calculations and reduce the amount of data. For further analysis, the mean value of these two-hour segments was calculated.

Linear mixed models were used to evaluate pigs’ preference behaviour (i.e., lying, feeding and active behaviours) in the four different pen compartments (i.e., three lighting conditions: 80 lx at 3000 K (pen C), 80 lx at 6500 K (pen D) and two pens (A and B) close to 0 lx at 0 K). Multiple comparisons were performed using the least squares means (LSMEANS) statement and the Tukey–Kramer adjustment. Statistical assumptions were checked using a graphical analysis of residuals focusing on the models’ distribution and homoscedasticity of errors. The lighting preference model included the lighting condition (factor with four levels: 3000 Kelvin at 80 lux (C), 6500 Kelvin at 80 lux (D), ~0 lux (A), and ~0 lux (B)), day of the experiment (factor with six levels: 1–6, i.e., 3 days/week for each batch), time of day (factor with 11 levels: 00:00, 02:00, 04:00, 06:00, 10:00, 12:00, 14:00, 16:00, 18:00, 20:00 and 22:00) and their interactions as fixed effects. This final model was calculated for each experimental week (i.e., weeks 1, 3 and 5) after F-tests for overall significance (*p* < 0.05) had been calculated. Mixed effect models (MIXED procedures) were used to examine the “lying”, “eating” and “active” behaviours of the piglets.

Variance components were estimated using the restricted maximum likelihood (REML) method. For the residual effects, heterogeneous residual variances were modelled.

A generalised linear mixed model (GLIMMIX procedure) was then used to determine the illuminance preference, i.e., the preference for light or darkness. For this purpose, the observations in both illuminated compartments (3000 Kelvin and 6500 Kelvin at 80 lux) were evaluated as one observation in brightness. Similarly, both darkened compartments were considered as one unit. The individual animal observations for each 2 h period were aggregated for each animal and considered as binomially distributed (x of 24 trials). The final model included the batch (factor with two levels: 1–2), time of day (factor with 11 levels: 00:00, 02:00, 04:00, 06:00, 10:00, 12:00, 14:00, 16:00, 18:00, 20:00 and 22:00) and the interaction between batch and time of day as fixed effects. Using the random int/subject = animal option, the animal was modelled as a random effect, and the random _residual_/subject = animal type = ar(1) option was used to model possible autocorrelations of repeated observations on the same animal over time. This approach had the advantage that there were no more convergence problems for week 1 and the same model fitted for all 3 weeks. Proportions under this binomial distribution were logit-transformed for the evaluation. Variance components were estimated using the maximum likelihood estimation method.

A chi-squared test was used to compare the distribution of cleanliness scores in each pen (illuminated with 80 lux and 3000 Kelvin (C), 80 lux and 6500 Kelvin (D) and ~0 lux and 0 Kelvin (A, B; A and B were grouped for the chi-square test)). The areas “drinking”, “eating” and “lying” were often not soiled or only slightly soiled. This would have resulted in too many classes being understudied in the evaluation scheme of the chi-squared test, which would have violated the requirements for the feasibility of the test (each cell of the contingency table must contain at least five expected values). Therefore, only the defaecating area was used to assess the cleanliness of the pens.

## 3. Results

### 3.1. Light and Darkness Preferences

A total of 57,024 observation time points (12 observations/hour × 22 h × 3 experimental days × 3 weeks × 2 batches × 12 animals; 28,512 observations for each batch) were recorded and analysed for the experiment in both batches. There were a total of 27,929 observations made under 80 lx (48.9% of all observations) and 29,095 observations (51.1% of all observations) made in the compartments close to 0 lx (Table 1).

With 79.0% of all observed behaviours, “lying” was the most frequently shown behaviour. The “eating” and “active” behaviours occurred in 5.4% and 15.6% of the cases, respectively.

**Figure 3:**
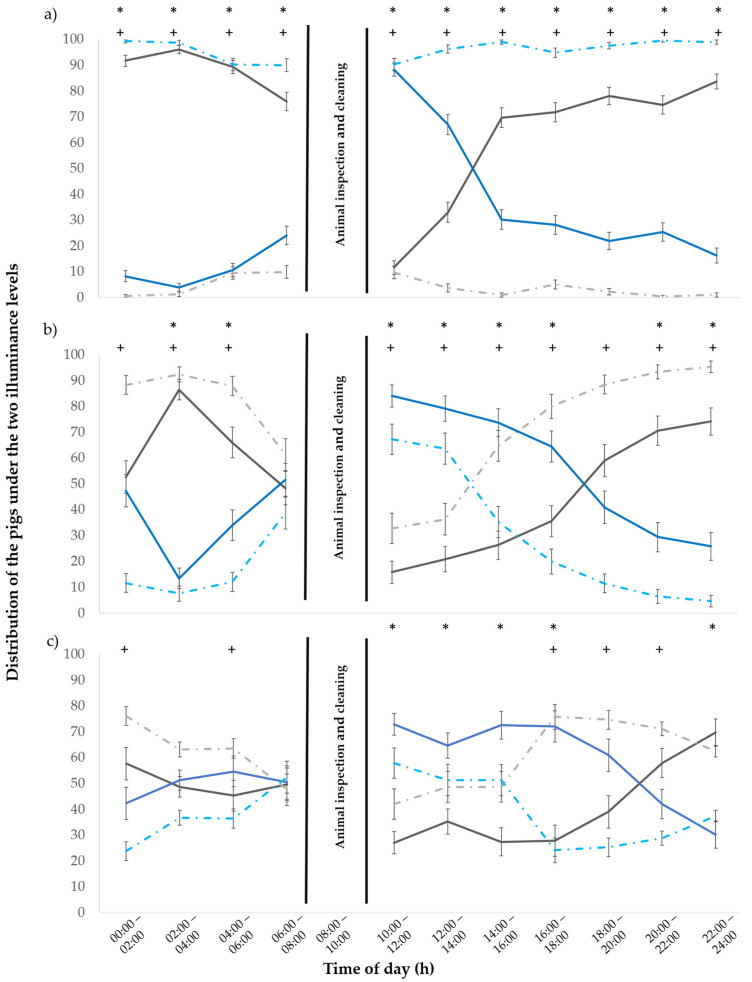
Distribution of the pigs (in %) throughout the day (24 h) under 80 lux (batch 1 dark blue, batch 2 light blue, dotted line) and almost 0 lux (batch 1 black, batch 2 light grey, dotted line). Illuminances during (**a**) the first, (**b**) third and (**c**) fifth experimental week. The error bars represent the standard error (±SE) of the mean. An asterisk (*, batch 1) and a plus (+, batch 2) shows a statistical difference with *p* < 0.050 between the illuminated and darkened compartments.

During the first experimental week, the batch, time of day and the interaction batch × time of day (*p* < 0.001) influenced the behaviour of the animals (Figure 3a). The piglets remained in the darkened compartments longer in the first batch (batch 1: 75% in the darkened pens vs. 25% in the illuminated pens; *p* < 0.001), while, in the second batch, they remained in the illuminated compartments more often (batch 2: 2% in the darkened pens vs. 98% in the illuminated pens; *p* < 0.001). In the first batch, the piglets preferred to stay in the darkened compartments in the morning (00:00 to 08:00) and evening (14:00 to 00:00). They spent late morning–midday (10:00 to 14:00) in the illuminated pens. During the second batch, piglets spent the most time in the illuminated pens without changing their preference. In the third week, the batch, time of day and the interaction batch × time of day (*p* ≤ 0.007) influenced the behaviour of the animals (Figure 3b). The piglets stayed more in the darkened pens (batch 1: 51% in the darkened pens vs. 49% in the illuminated pens, *p* < 0.9; batch 2: 80% in the darkened pens vs. 20% in the illuminated pens, *p* < 0.001). From 00:00 to 06:00 and from 15:00 to 00:00, the piglets preferred to stay in the darkened pens. From 10:00 to 14:00, the piglets preferred the 80 lx pens.

In the fifth experimental week, the batch, time of day and the interaction batch × time of day (*p* ≤ 0.01) influenced the behaviour of the animal (Figure 3c). Preference behaviour differed between the batches. For the first batch, piglets stayed more in the illuminated pens (batch 1: 43% in the darkened pens vs. 57% in the illuminated pens, *p* = 0.2). Piglets in the second batch stayed more in the darkened pens (batch 2: 61% in the darkened pens vs. 39% in the illuminated pens, *p* = 0.018).

### 3.2. Colour Temperature Preference

Out of 57,024 observations, 51.02% were made in the darkened pens with 0 K (23.9% in pen A, 27.1% in pen B), 32.1% under 80 lx and a colour temperature of 3000 K and 16.9% under 80 lx and 6500 K (Table 2).

The most frequently shown behaviour was “lying” with 79.0%, followed by “active” with 15.7% and “eating” with 5.3%. Overall, the “lying” behaviour was observed more often in the darkened pens (A + B), followed by the 3000 K illuminated pen (C) and the least in the 6500 K illuminated pen (D). The “eating” behaviour was equally recorded in all pens. The animals showed the “active” behaviour under all lighting conditions, with a relatively even distribution.

**Figure 4:**
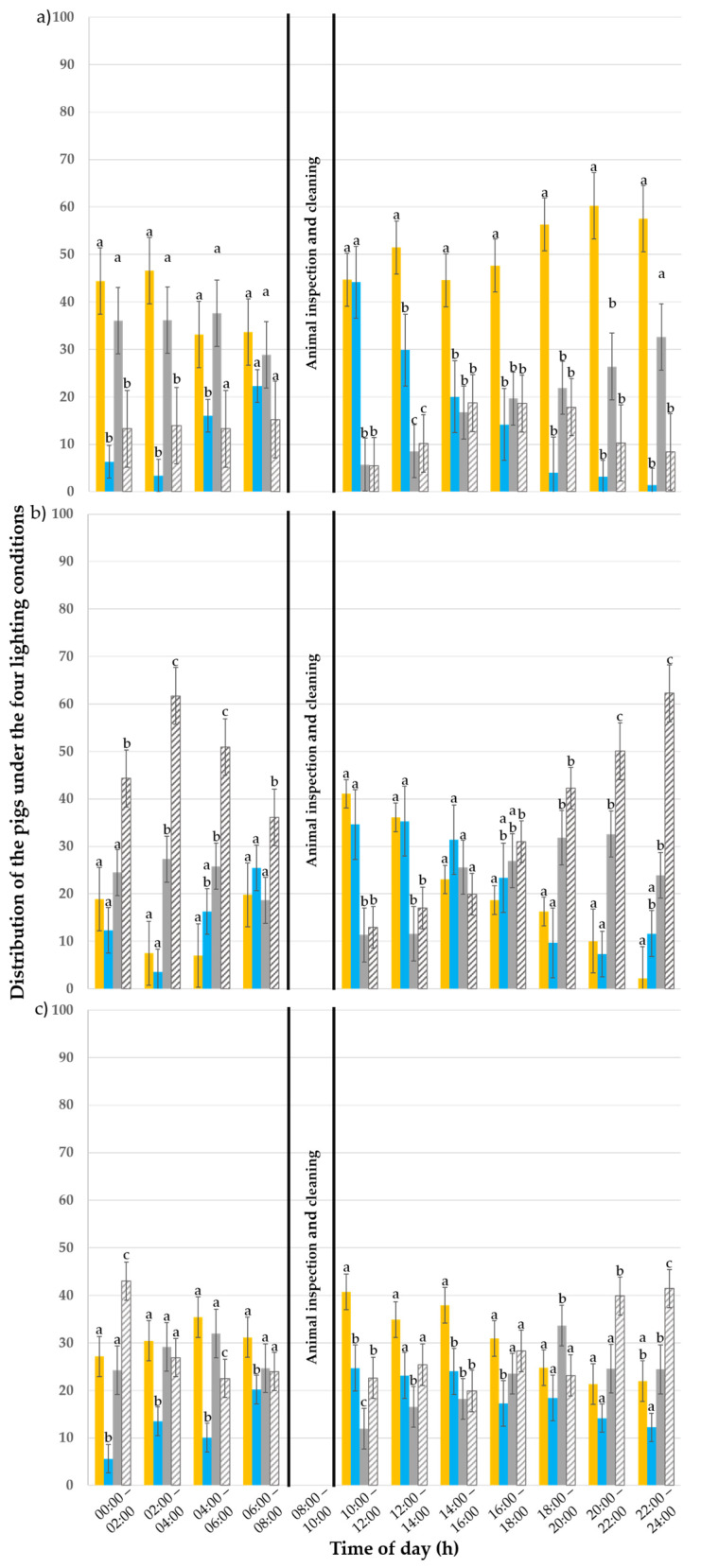
Distribution of the pigs (%) throughout the day (24 h) under 80 lux and 3000 Kelvin (yellow), 80 lux and 6500 Kelvin (blue) and 0 lux pens (grey and striped grey for the darkened pens A and B, respectively) during the (**a**) first, (**b**) third and (**c**) fifth experimental week. The error bars represent the standard error (±SE) of the mean. Different letters above the bars indicate a statistical difference with *p* < 0.050.

During the first experimental week, the day of observation (*p* = 0.360) and time of day (*p* = 1) had no influence on the behaviour of the pigs (Figure 4a). The lighting condition and the interactions day × lighting condition and time of day × lighting condition (*p* < 0.001) had an influence. From 12:00 to 22:00, the colour temperature of 3000 K was preferred over all other lighting conditions (*p* ≤ 0.010).

In the third week, the animals were most frequently observed in the darkened compartments (Figure 4b). The day of observation (*p* = 0.459) and time of day (*p* = 1) had no influence on the pigs’ behaviour, but the lighting condition and its interactions between time of day × lighting conditions and day × lighting conditions had an influence (*p* ≤ 0.010). From 20:00 until 06:00, the pigs showed a preference for darkness (*p* ≤ 0.020), whereas the illuminated compartments were favoured from 10:00 to 14:00 (*p* ≤ 0.030).

In the fifth experimental week, the animals were more equally distributed under the different lighting conditions (Figure 4c). The day of observation (*p* = 0.460) and time of day (*p* = 1) had no influence on the pigs’ behaviour. The lighting condition and its interactions with the day and time of day (*p* < 0.001) had an influence. At 10:00 and 14:00, the animals showed a clear preference for the colour temperature of 3000 K (*p* ≤ 0.020), whereas, in the evening hours from 20:00 onwards until 02:00, they preferred the dark pen B (*p* ≤ 0.010).

#### 3.2.1. “Lying” Behaviour

Out of a total of 57,024 observations, 45,080 (79% of all observations) thereof were made for “lying” pigs. In the first week, 15,581 pigs were observed “lying”; in the second week, 14,822 observations were made; and, in the third week, a total of 14,682 observations were made. Table 3 shows how the observations made for “lying” were distributed across the different lighting situations.

**Figure 5:**
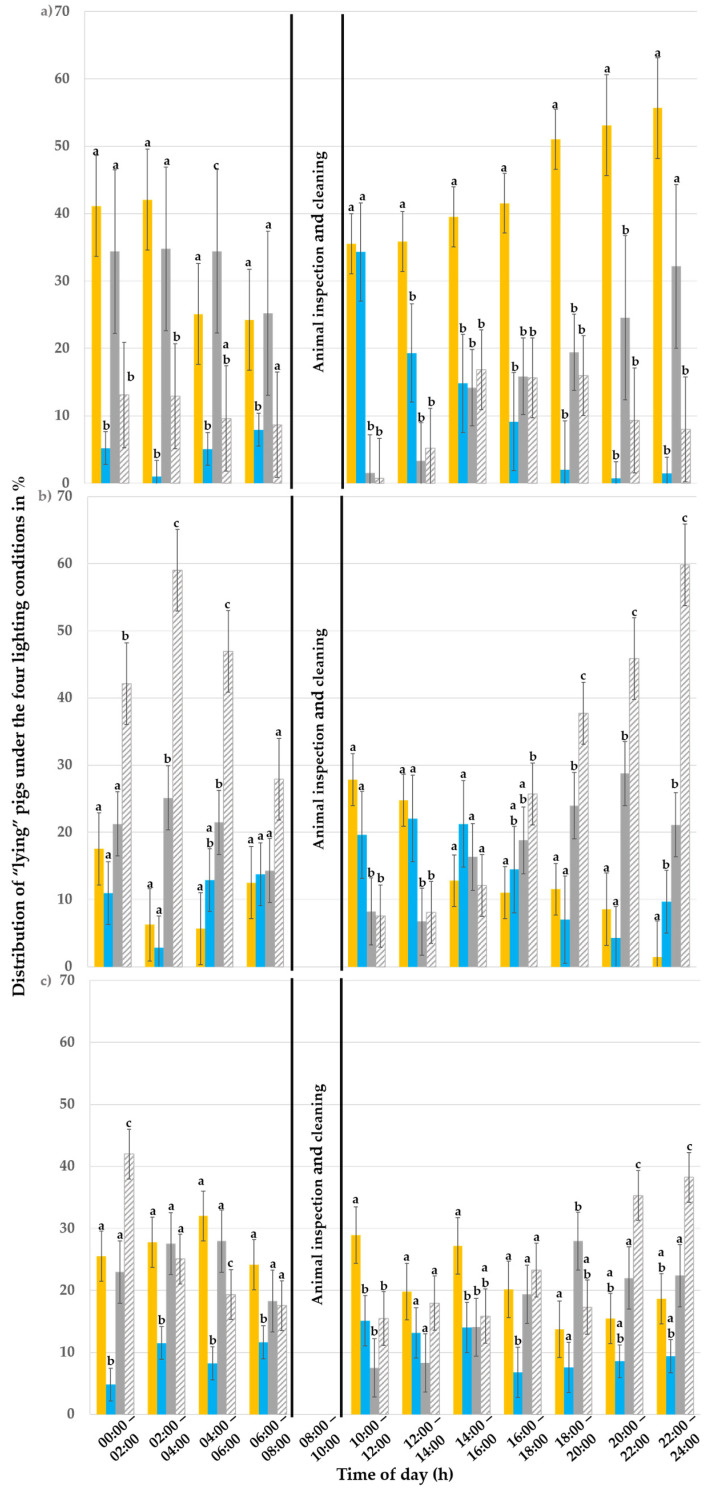
Distribution of the “lying” pigs (%) throughout the day (24 h) under the 80 lux and 3000 Kelvin (yellow; pen C), 80 lux and 6500 Kelvin (blue, pen D), and 0 lux pens (grey and striped grey for the darkened pens A and B, respectively) during the (**a**) first, (**b**) third and (**c**) fifth experimental week. The error bars represent the standard error (±SE) of the mean. Different letters above the bars indicate a statistical difference with *p* < 0.050.

In the first week (Figure 5a), the lighting condition (*p* < 0.001) and its interactions with the day of observation (*p* < 0.001) and with the time of day (*p* = 0.002) had an influence on the pigs’ lying behaviour. However, the day of observation (*p* = 0.410) and time of day alone (*p* = 0.780) had no effect. Between 14:00 and 22:00, the pigs preferred the 3000 K illuminated pen to all other pens for lying (*p* ≤ 0.046). The colour temperature of 3000 K was always preferred to 6500 K (*p* ≤ 0.046), except between 10:00 and 14:00 (*p* ≥ 0.050). From 22:00 to 06:00, the pigs showed no preference for lying between the dark pen A and the 3000 K illuminated pen C (*p* ≥ 0.142), while both were preferred to the 6500 K illuminated pen D. At 10:00, both illuminated pens were preferred to the dark pens for the “lying” behaviour.

In the third week (Figure 5b), the day of observation (*p* = 0.790) and time of day (*p* = 0.110) had no effect on the pigs’ lying behaviour, whereas the lighting condition (*p* < 0.001) and its interactions with the day (*p* < 0.001) and with the time of day (*p* < 0.001) had an effect. Dark pen B was used most often to show “lying” behaviour between 18:00 and 06:00 compared to the other pens (*p* ≤ 0.045). The 3000 K illuminated pen was preferred to lie down in between 10:00 and 14:00 compared to the darkened pens (*p* ≤ 0.005) but not to the 6500 K illuminated pen (*p* ≥ 0.175). At 06:00 and 14:00 no pen was preferred to lie down in.

In the fifth experimental week (Figure 5c), the day of observation had no effect on the pigs’ lying behaviour (*p* = 0.990), whereas the time of day, the lighting condition and its interactions with the day of observation and with the time of day had an effect (*p* ≤ 0.020). The 80 lx and 3000 K lighting condition was preferred to all others at 10:00 (*p* ≤ 0.030) and additionally preferred to the 6500 K lighting condition between 14:00 and 18:00 and from 00:00 to 08:00 a.m. (*p* ≤ 0.030). However, darkened pen B was preferred to lie down in to all others between 20:00 and 02:00 (*p* ≤ 0.039). Between 12:00 and 14:00, no preference was shown for lying down for any of the lighting conditions.

#### 3.2.2. “Eating” Behaviour

Out of a total of 57,024 observations, 2998 (5.3% of all observations) thereof were made for “eating” pigs. In the first week, 766 eating pigs were observed; in the second week, 1107 observations were made; and, in the third week, 1125 observations were made. Table 4 shows how the observations were distributed across the different lighting situations.

**Figure 6:**
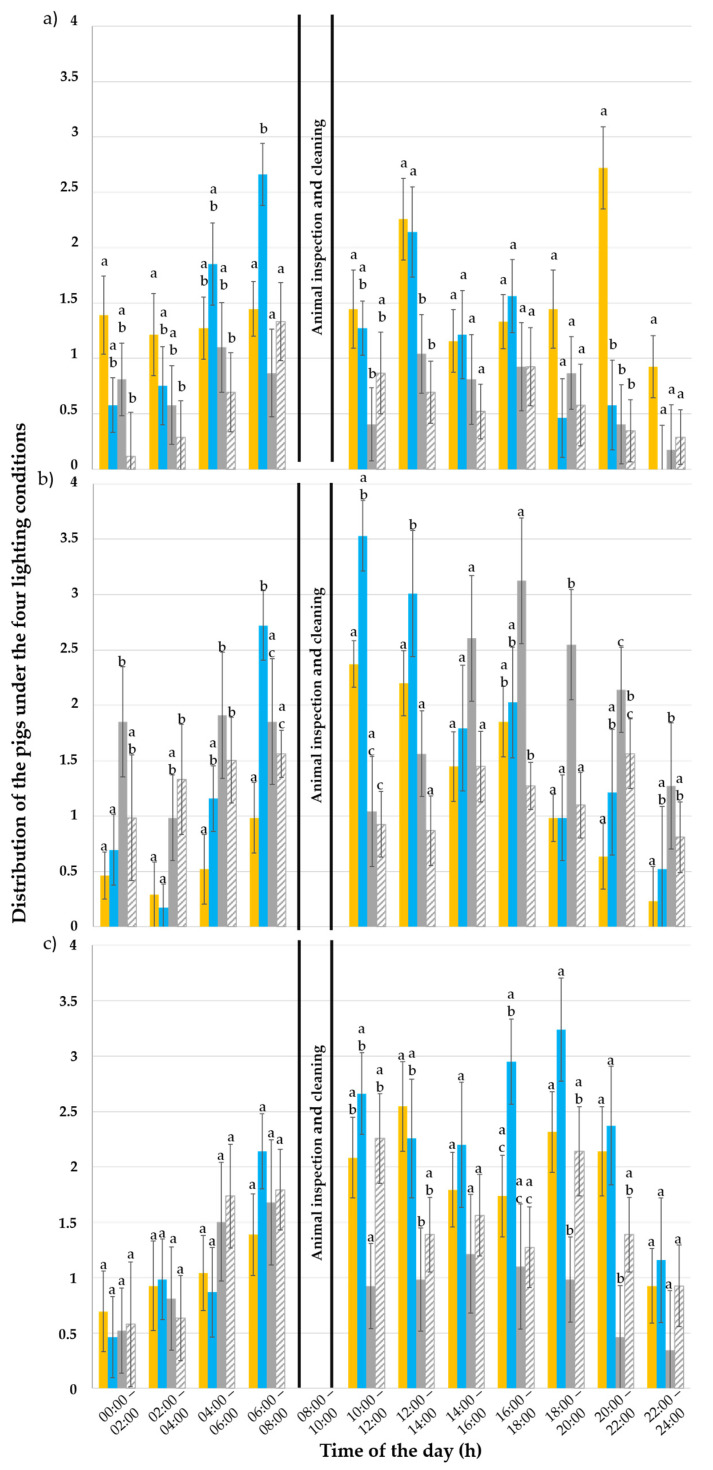
Distribution of the “eating” pigs (%) throughout the day (24 h) under the 80 lux and 3000 Kelvin (yellow; pen C), 80 lux and 6500 Kelvin (blue, pen D), and 0 lux pens (grey and striped grey for the darkened pens A and B, respectively) during the (**a**) first, (**b**) third and (**c**) fifth experimental week. The error bars represent the standard error (±SE) of the mean. Different letters above the bars indicate a statistical difference with *p* < 0.050.

In the first experimental week (Figure 6a), the day of observation (*p* < 0.001), time of day (*p* < 0.001), lighting condition (*p* < 0.001) and its interactions with the day of observation (*p* < 0.001) and with the time of day (*p* = 0.022) affected the “eating” behaviour of the pigs. During the day, more pigs were observed eating in the illuminated pens compared to the darkened ones (*p* ≤ 0.010). The “eating” behaviour was, however, not exhibited more often in either of the illuminated pens (*p* = 0.190) nor between the two darkened pens A and B (*p* = 0.780). At 06:00, the pigs tended to eat under the 6500 K light (*p* ≤ 0.018). At 12:00, the animals were seen eating more often in the lit pens (*p* ≤ 0.034), while, at 20:00, more pigs were observed eating under the 3000 K and 80 lx lighting condition compared to all the other lighting conditions (*p* < 0.001).

In the third week (Figure 6b), the time of day (*p* < 0.001), lighting condition (*p* < 0.001) and its interaction with the day of observation (*p* < 0.001) and with the time of day (*p* < 0.001) had an effect on the pigs’ “eating” behaviour, whereas the day of observation had no effect (*p* = 0.580). Pigs were not observed more often under either the illuminated or darkened pens (*p* ≥ 0.080). Nevertheless, they ate more frequently under the 6500 K colour temperature compared to the 3000 K one (*p* = 0.020) as well as in the darkened pen A compared to the darkened pen B (*p* < 0.001) and 3000 K one (*p* < 0.001). They did not eat more often in the 3000 K pen C compared to the darkened pen B (*p* = 0.870) or in the 6500 K pen D compared to the darkened pens A and B (*p* ≥ 0.083). At 0:00, more pigs ate in the darkened pen A compared to pen C and pen D (*p* ≤ 0.008). At 02:00, pigs were observed eating more often in darkened pen B compared to the illuminated pens (*p* = 0.008) but not compared to the darkened pen A (*p* = 0.430). At 06:00, more pigs ate in the pen illuminated at 6500 K (pen D) compared to all other pens (*p* ≤ 0.040). At 10:00, pigs ate more frequently in the lit pens (pen C and pen D) (*p* ≤ 0.036), whereas, at 18:00, they displayed the most eating behaviour in the darkened pen A (*p* ≤ 0.039).

In the fifth week (Figure 6c), the time of day, lighting condition and interaction between lighting condition × day of observation had an effect on pigs’ “eating” behaviour (*p* < 0.001). The day of observation (*p* = 0.310) and the interaction of the time of the day × lighting condition (*p* = 0.140) had no effect. Pigs were not observed eating more often under the 3000 K than the 6500 K illuminated pen (*p* = 0.330) and under the 3000 K than the darkened pen B (*p* = 0.780). The 6500 K pen, however, was more frequently used to display eating behaviour than the darkened pens A and B (*p* ≤ 0.040), whereas the darkened pen A was the least frequently used over all others for eating (*p* ≤ 0.030).

#### 3.2.3. “Active” Behaviour

Out of a total of 57,024 observations, 8946 (15.7% of all observations) were made of pigs being “active”. In the first week, 2661 active pigs were observed; in the second week, 3084 observations were made; and, in the third week, 3201 observations were made. Table 5 shows how the observations were distributed across the different lighting situations.

**Figure 7:**
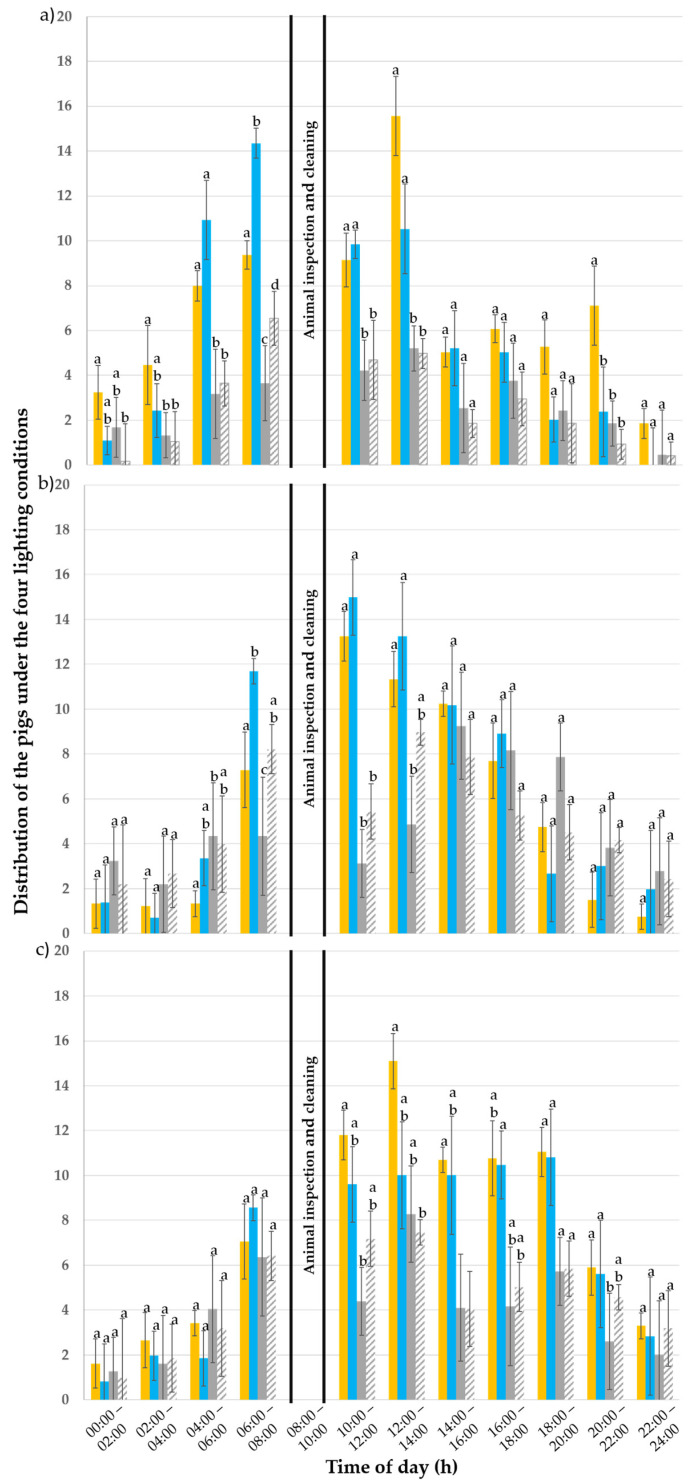
Distribution of the “active” pigs (%) throughout the day (24 h) under the 80 lux and 3000 Kelvin (yellow; pen C), 80 lux and 6500 Kelvin (blue, pen D) and 0 lux pens (grey and striped grey for the darkened pens A and B, respectively) during the (**a**) first, (**b**) third and (**c**) fifth experimental week. The error bars represent the standard error (±SE) of the mean. Different letters above the bars indicate a statistical difference with *p* < 0.050.

During the whole trial (batch one and two), the pigs showed the highest activity between 06:00 a.m. (early morning hours) and 18:00. In the evening hours and during the night (from 20:00 to 04:00 a.m.), the pigs showed little to no active behaviour. In the first week (Figure 7a), the day of observation (*p* < 0.001), time of the day (*p* < 0.001), lighting condition (*p* < 0.001) and its interactions with the day of observation (*p* < 0.001) and with the time of the day (*p* < 0.002) affected the “active” behaviour of the pigs. The animals favoured the illuminated pens (80 lx with 3000 K (C) or 6500 K (D)) over the darkened ones (pens A and B; *p* < 0.001). However, they made no difference between the 3000 K (C) and 6500 K illuminated pens (*p* = 0.470) and neither between the darkened pens A and B (*p* = 0.770). At 04:00 a.m., the 3000 K (C) and 6500 K (D) pens were preferred to the darkened pens A and B (*p* ≤ 0.002). At 06:00 a.m. the 3000 K pen (C) was preferred over the other pens (*p* ≤ 0.038) and the 6500 K (D) pen to the A and B darkened pens (*p* < 0.001). At 10:00 a.m. and 12:00, the illuminated pens (C and D) were preferred to the darkened pens A and B (*p* ≤ 0.040). At 20:00 the 3000 K pen (C) was preferred to the 6500 K (D) and the darkened pens A and B (*p* = 0.027).

In the third experimental week (Figure 7b), the day of observation (*p* = 0.060), lighting condition (*p* = 0.110) and its interaction with the day of the observation (*p* = 0.110) did not influence pigs’ “active” behaviour. The time of the day (*p* < 0.001) and its interaction with the lighting condition (*p* < 0.001) had an influence. The pigs were equally active in all pens throughout the day and had no preference for one light situation (*p* ≤ 0.280). Between 10:00 a.m. and 12:00, most pigs were active in the illuminated pens (*p* < 0.001) but did not prefer one colour temperature over the other (*p* = 1). At 12:00, both illuminated pens (C and D) were preferred over darkened pen A (*p* ≤ 0.006).

In the fifth week (Figure 7c), the day of observation (*p* = 0.500) and the interactions between the lighting condition and the day of observation (*p* = 0.140) and the interaction between the time of day and the lighting condition (*p* = 0.370) did not influence the pigs’ “active” behaviour. The lighting condition (*p* < 0.001) and time of day (*p* < 0.001) had, however, an influence. Throughout the day, the pigs preferred the illuminated pens (C and D) more than the darkened ones (A and B; *p* ≤ 0.003). Nonetheless, during the day, no preference was observed for one colour temperature over the other (*p* = 0.270) or between the two darkened pens A and B (*p* = 0.370). The animals were active in the early morning hours from 06:00 until 18:00 in the evening. They were more active between 10:00 and 18:00 in the illuminated pens (C and D).

### 3.3. Pen Compartment Cleanliness

The lighting condition affected the pen cleanliness scores throughout the experiment (x^2^_3_ = 135.27; *p* < 0.001; Table 6).

Darkened pens A and B, which were grouped together, showed the lowest overall soiling. They received a score of 0 (not soiled) 5 times, score 1 (very slightly soiled, i.e., visible individual piles of faeces) 36 times, score 2 (slightly soiled, i.e., slightly visible soiling with faeces covering less than 25% of the floor) 49 times and score 3 (moderately soiled, i.e., medium visible soiling with faeces covering more than 25% and up to 50% of the floor) only 3 times. Pen C was moderately soiled, with 1 observation with score 0, 12 with score 1, 50 with score 2 and 6 with score 3. Pen D was clearly the most soiled, receiving a score of 3 in 47 observations. Lower scores were rare for Pen D, with only 1 observation each with score 0 and score 1 and 19 observations with score 2.

## 4. Discussion

This study’s objective was to determine whether and how four-week-old weaned piglets’ preference behaviour for lying, eating, being active and defaecating changes depending on the lighting condition. During a five-week experiment, the behaviour of piglets and the cleanliness of the pens were recorded and evaluated under four different lighting conditions: 80 lx and 3000 K (pen C), 80 lx and 6500 K (pen D) and darkened close to ~0 lx and 0 K (pens A and B).

The piglets initially spent most of their time lying in the illuminated pens (preferably under 3000 K), then reversed their preference to stay for the darkened pens before no longer showing any preference to stay in the last experimental week. Overall, pigs were more active in the illuminated pens and soiled the 80 lx and 6500 K pen more often than the other pens. Both the light intensity and colour temperature therefore seem to have an effect on the behaviour of the pigs.

During the first experimental week, piglets did not show a clear preference to stay in dark or illuminated compartments. In the first batch, in particular, the animals behaved differently than in the second batch and showed a preference for the darkened compartments, whereas the piglets in the second batch tended to prefer the illuminated pens. There may be various reasons for this, such as the age of the piglets or the conditions in the farrowing pen [23,24]. Unfortunately, this explanation remains to be proven, as, in this case, the conditions in the farrowing pens were not known and could not be controlled. In studies by Tanida et al. [22], one-week-old suckling piglets showed a tendency to actively move towards light areas and actively move away from dark areas. In contrast, Parfet et al. [35] observed that newborn piglets are more attracted to darker areas. In their motivation study with 14-week-old pigs, Baldwin and Meese [36] found that the pigs strongly preferred light to darkness and spent at least 72% of their time in the light.

The exact age of the animals could not be determined in this study; according to the producer, all animals delivered were of the same age. However, the different ages of the animals could be a possible explanation for the differences seen within the batches. The piglets showed a preference for the 3000 K colour temperature compared to 6500 K, with almost 50% of the pigs staying in the 80 lx and 3000 K lit pen and 25% in the 80 lx and 6500 K pen. This aligns with our previous findings [2]. However, why the pigs preferred the colour temperature of 3000 K remains unclear. It is also known from various studies with mice and broilers that the animals also preferred reddish light to other light colours [30,31,36,37,38]. Piglet nests are commonly equipped with red heat lamps (emitting a reddish light) that provide a familiar atmosphere for the piglets to orient themselves. Christison et al. [23] and Phillips et al. [24] found that familiar environmental factors such as brightness and odour influence piglets and their preferences. This could be a possible explanation why piglets preferred warm colour temperatures as they are similar to piglet nests (emitting a reddish light). As already described, it was unfortunately not possible to assess the conditions on the initial commercial housing site.

During the third week of the trial, the pigs spent more time in the dark pens, reversing the original preference of the second batch for staying most of their time in the lit pens. Hacker et al. [39] also found that pigs given a continuous choice between a lit and an unlit pen spent 75% of the day in the darkness. In this study, it was observed that the pigs preferred to stay in the lit pens during the day (between 06:00 and 14:00). During this time, they had no preference for one specific colour temperature. From late afternoon to early morning (16:00 to 04:00), the piglets moved to the darkened pens. Sambraus [40] described a 12 h daily rhythm in pigs that was maintained by feeding and grooming times, even in the absence of natural light. This 12 h rhythm can also be observed in wild boars, where they spend most of the day foraging and use the night to rest [38,39].

In the fifth week of the experiment, the pigs no longer showed any preference for a specific lighting condition (3000 K (pen C) with 30.6% vs. 6500 K (pen D) with 16.7% vs. dark pen A with 23.9% vs. dark pen B with 28.8%). Piglets in the first batch also showed no preference for illuminated or darkened pens, whereas piglets in batch two preferred to stay in the darkened pens.

These results are consistent with the observations in our two earlier studies [2,3], in which the piglets showed no preference for lighting in the later weeks, although the adaptation to the course of the day remained visible. It can be assumed that other factors, such as the amount of space available or spatial proximity, had a greater influence on the piglets’ choice of pen than solely the light intensity.

Lying was the most frequently observed behaviour, as often reported in the literature [40,41,42]. In the first experimental week, the animals were most often observed lying down in the illuminated pen with 80 lx and 3000 K but were also seen lying in the darkened pens. A possible explanation for this is given by Taylor et al. [21], who observed similar results and suspected that pigs tend to prefer a series of lighting conditions over a single one. However, this behaviour was not reported by Zeng et al. [8]. In the third week, the piglets’ general and lying down preferences changed to the darkened pens. The preference to use the illuminated pens at midday was also reflected in the lying behaviour and followed the aforementioned daily rhythm of the pigs. By the fifth week, there was no clear preference for light intensity. An age effect, in which pigs change their preference, has been previously observed in other studies [2,3]. Taylor et al. [21] and Zeng et al. [8] were unable to demonstrate this changing preference with the age of the animals. One explanation for the observed decrease in light preference with increasing weeks could therefore be the relative decrease in space availability due to the increasing size of the piglets as they grew (in this study, from 2.02 kg weight/m^2^ per pen at the beginning to 6.1 kg weight/m^2^ per pen at the end of the experiment).

The “eating” behaviour was the least frequently observed behaviour. This was probably due to the time sampling observation method (i.e., one picture every five minutes) and the fact that conventionally housed pigs spend a reduced amount of time eating, i.e., 28 to 37 min out of 15 h of observation time [41] or 140 min per day [42]. In our study, no effect of the lighting condition on the eating behaviour of the pigs was observed, which is consistent with Taylor et al. [21] and Zeng et al. [8], who found no effect of the light intensity on pigs’ feeding behaviour.

Pigs were observed to be active mainly during the natural light phase of the day, though this behaviour only represented a relatively small percentage (15.6%) of the total recorded behaviours. In our previous studies [2,3] as well as in Zaludik [43], for pigs kept on partially or fully slatted floors, even lower activity levels were reported (i.e., pigs being active during 8–11% of the day). The higher percentage of activity observed in the present study can be explained by the larger space available (1.39 m^2^/pig versus 0.5 m^2^/pig) and the fact that the different lighting conditions probably stimulated the pigs’ natural exploratory and play behaviour. Throughout the experiment, no preferred light condition could be found in which the animals were more active, which corresponds to the statements of Taylor et al. [21], who suggested offering the pigs a variety of enrichment options to satisfy their natural interests. Similar results were found in the study by Scaillierez et al. [44]. Piglets raised in an environment with a light gradient (71 lx in the front of the pen and 330 lx in the back of the pen; on average, 198 lx) showed an increase in positive social interactions. Over the five-week observation period, an increase in piglet activity was recorded during the early morning hours between 06:00 and 08:00, followed by a decline in activity in the evening between 18:00 and 20:00. A similar temporal pattern was observed in feeding behaviour, while the number of animals lying down significantly decreased during these intervals. These time-dependent behavioural patterns can be attributed to the pigs’ circadian rhythm, which remains intact even in the absence of natural daylight [45,46]. It is known from other studies that pigs adapt their behaviour to the occurrence of external repetitive events [47,48]. The routine procedures of animal inspection and barn cleaning, which always took place at the same time, may have acted as such external time cues, which were perceived by the piglets and had a synchronising effect on their behaviour.

Throughout this study, a daily influence on the behaviour of the piglets was observed. Pigs reacted to external influences, such as noises and voices from outside the test room. Although the days (Tuesday, Saturday and Sunday) on which the pigs’ behaviour were studied were chosen to minimise these negative effects, not all disturbances could be avoided. It was not possible to analyse retrospectively which noises affected the animals and to what extent. For future experiments, the volume of the noises outside the test room could be recorded and correlated with the behaviour of the animals. Analysing more days may have led to less influence of the particular day. It would also be conceivable to repeat the experiment at a different location to minimise the effect of external influences.

Regarding the cleanliness of the pen, the illuminated pen with 80 lx and a colour temperature of 6500 K was more soiled than the darkened pens or the pen illuminated with 80 lx and 3000 K. This is consistent with previous studies that reported that brightly lit compartments were more soiled than darkened ones [2,3,21,49,50]. It has been demonstrated that pigs do not defaecate where they rest [48,51,52,53]. Consequently, it can be concluded that the pigs avoid soiling the pens where they rested the most. However, it is not possible to clearly determine which effects lead to the choice of lying position or defaecation [48].

## 5. Conclusions

This study demonstrates that pigs do not have a fixed preference for a specific lighting condition (continuous darkness or 80 lx LED light at 3000 K or 6500 K) but adjust their behaviour according to light exposure. Initially, they preferred lying in the illuminated 3000 K pens but, from the third week onwards, they increasingly preferred the darker pens. By the end of the study, no overall preference for light or dark was observed, though pigs still favoured 3000 K for resting. Their behaviour followed a circadian rhythm, with more time spent in darkness during the evening and in illuminated areas from midday onwards.

Lighting had no significant impact on activity or feeding behaviour but influenced pen cleanliness. Pens with 80 lx and 6500 K were more soiled, likely due to pigs avoiding them for resting.

This study should be considered a pilot study designed to address the initial question of whether pigs can distinguish between three different lighting situations as indicated by behavioural adaptations to these conditions. Further studies or additional trials are nevertheless necessary to confirm our results with regard to the specific study design as well as to the number and age of the animals tested.

These findings suggest that lighting can be used to influence pen usage, potentially helping to create functional areas that align with pigs’ natural behaviours. Further research should explore the impact of different colour temperatures and determine an optimal upper limit for lighting intensity, as inadequate lighting could negatively affect animal welfare. Adapting lighting conditions in pig housing may enhance well-being by providing environments that support natural behavioural patterns.

## Figures and Tables

**Table 1:** Distribution of the observed behaviours (in %) under 80 lux (lx) and close to 0 lx illuminance levels for both batches (B1 for batch 1/B2 for batch 2).

	Observed Behaviours (%)							
Illuminance Level	“Lying”		“Eating”		“Activity”		Total	
	B1	B2	B1	B2	B1	B2	B1	B2
80 lx	33.3	38.7	2.5	3.5	8.9	11.1	44.6	53.3
~0 lx	47.3	38.8	2.9	1.7	5.2	6.2	55.4	46.7
total	80.6	77.5	5.4	5.2	14.1	17.3		

**Table 2:** Distribution of the behaviours observed (in %) under 3000 Kelvin (K), 6500 K or close to darkness (0 K) for both batches (B1 for batch 1/B2 for batch 2) in pens A–D.

	Observed Behaviours (%)							
Colour Temperature	“Lying”		“Eating”		“Activity”		Total	
	B1	B2	B1	B2	B1	B2	B1	B2
3000 K (C)	22.1	28.7	1.1	1.7	4.9	5.6	28	36.1
6500 K (D)	11.2	10.0	1.4	1.8	4.0	5.5	16.7	17.2
0 K (A)	25	15.3	1.5	0.9	2.8	2.7	29.2	18.8
0 K (B)	22.3	23.5	1.4	0.8	2.4	3.6	26.1	27.9
Total	80.6	77.5	5.4	5.2	14	17.4		

**Table 3:** Overview of estimated “lying” pigs per lighting situation.

	Colour Temperature			
Week	80 lux		~0 lux	
	3000 Kelvin (Pen C)	6500 Kelvin (Pen D)	0 Kelvin (Pen A)	0 Kelvin (Pen B)
1	40.45	9.18	21.8	10.54
2	12.71	12.61	18.73	33.84
3	23.03	10.06	19.85	24.3

**Table 4:** Overview of estimated “eating” pigs per lighting situation.

	Colour Temperature			
Week	80 lux		~0 lux	
	3000 Kelvin (Pen C)	6500 Kelvin (Pen D)	0 Kelvin (Pen A)	0 Kelvin (Pen B)
1	1.51	1.19	0.73	0.61
2	1.09	1.62	1.89	1.21
3	1.59	1.94	0.95	1.42

**Table 5:** Overview of estimate “active” pigs per lighting situation.

	Colour Temperature			
Week	80 lux		~0 lux	
	3000 Kelvin (Pen C)	6500 Kelvin (Pen D)	0 Kelvin (Pen A)	0 Kelvin (Pen B)
1	6.83	5.80	2.76	2.64
2	5.52	6.55	4.91	5.06
3	7.58	6.59	4.06	4.52

**Table 6:** Overview of pen soiling level.

	Pen Soiling Level (Number of Observations Per Score)			
Pen	Score 0	Score 1	Score 2	Score 3
A + B	5	36	94	3
C	1	12	50	6
D	1	2	19	47

## Data Availability

Data supporting the findings of this study, including the original survey instruments and related materials, are not publicly available due to privacy and confidentiality considerations but can be obtained from the corresponding author upon reasonable request.
